# Photic Entrainment of the Circadian System

**DOI:** 10.3390/ijms23020729

**Published:** 2022-01-10

**Authors:** Anna Ashton, Russell G. Foster, Aarti Jagannath

**Affiliations:** Nuffield Department of Clinical Neurosciences, Sleep and Circadian Neuroscience Institute (SCNi), University of Oxford, Oxford OX1 3QU, UK; anna.ashton@ndcn.ox.ac.uk

**Keywords:** circadian, clock, light, entrainment, SCN, zeitgeber

## Abstract

Circadian rhythms are essential for the survival of all organisms, enabling them to predict daily changes in the environment and time their behaviour appropriately. The molecular basis of such rhythms is the circadian clock, a self-sustaining molecular oscillator comprising a transcriptional–translational feedback loop. This must be continually readjusted to remain in alignment with the external world through a process termed entrainment, in which the phase of the master circadian clock in the suprachiasmatic nuclei (SCN) is adjusted in response to external time cues. In mammals, the primary time cue, or “zeitgeber”, is light, which inputs directly to the SCN where it is integrated with additional non-photic zeitgebers. The molecular mechanisms underlying photic entrainment are complex, comprising a number of regulatory factors. This review will outline the photoreception pathways mediating photic entrainment, and our current understanding of the molecular pathways that drive it in the SCN.

## 1. Introduction

The lives of most organisms on Earth, from bacteria to humans, are governed by the daily environmental changes that occur across the day/night cycle. In response, life has evolved an internal timing system, the circadian clock, to anticipate these changes and fine-tune physiology and behaviour to the varied demands of day and night.

**Figure 1:**
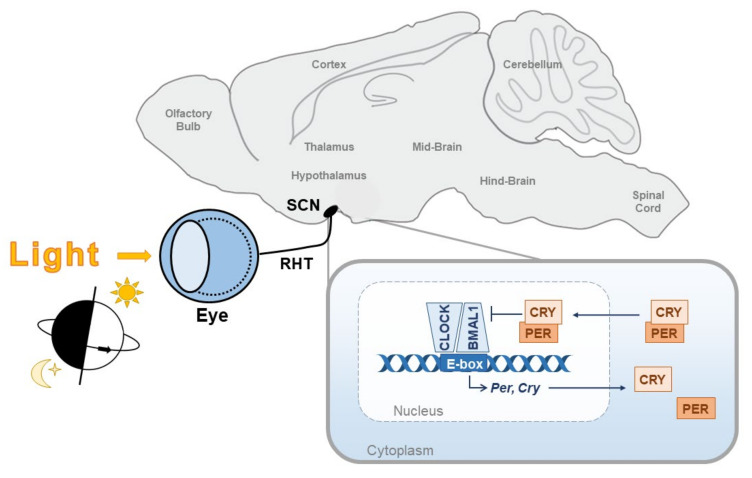
The circadian clock. The core circadian clock consists of a molecular transcriptional-translational feedback loop in which the transcription factors, CLOCK and BMAL1, heterodimerise and induce expression of the core clock genes, *Per* and *Cry*, via E-box response elements. PER and CRY then feedback onto CLOCK and BMAL1 by inhibiting their transcriptional activity. This feedback loop cycles with a period of around 24 h, therefore it must be continually readjusted to be aligned with the external world. The primary time cue for this is the daily light/dark cycle. Light information is transmitted via the retinohypothalamic tract (RHT) directly to the master clock in the suprachiasmatic nucleus (SCN).

The core circadian clock consists of an autoregulatory transcriptional-translational feedback loop (TTFL) whereby the transcription factors CLOCK and BMAL1 heterodimerise to drive expression of *Per* and *Cry* genes via E-box response elements [1]. PER and CRY proteins, within a protein complex, then feedback to suppress their transcription by inhibiting CLOCK:BMAL1 activity (Figure 1). The degradation of PER and CRY reverses this inhibition and the TTFL restarts. The result is a near 24 hour (h) oscillation in PER/CRY production and breakdown. It is now known that this core TTFL is at the centre of a complex network of additional feedback loops, which interact to regulate the precision and stability of the circadian clock (reviewed in [2]).

In humans and mice, the endogenous circadian clock cycles with a period slightly greater or less than 24 h, respectively. As a result, the clock must be continually readjusted to remain in alignment with the external world. This is achieved through a process termed entrainment using external time cues called “zeitgebers”. In mammals, entrainment begins by adjusting the phase of the master clock in the suprachiasmatic nuclei (SCN). The SCN in turn entrains the peripheral clocks throughout the body into a global alignment of the circadian system. In mammals, including humans, the primary zeitgeber is the changing light environment at dawn and dusk, which is transmitted directly to the SCN [3,4] (Figure 1). Within the SCN, light information is integrated with signals from a range of other non-photic zeitgebers including food, temperature and sleep, to align the biological and environmental day [5].

Such an alignment allows organisms to deliver the correct materials, in the correct concentration to the correct organ systems at the optimal time of day. This “fine-tuning” of biology is essential for survival. Without entrainment of the circadian system all “fine-tuning” is lost and physiology and behaviour drifts into chaos, termed “internal desynchrony”. Despite the critical importance of entrainment, the mechanisms that drive these pathways remain only partially understood. This review will outline our current understanding of the molecular mechanisms underlying photic entrainment, and discuss recent advances in how light signals are integrated with other non-photic zeitgebers.

## 2. Phase Shifting of the Clock

**Figure 2:**
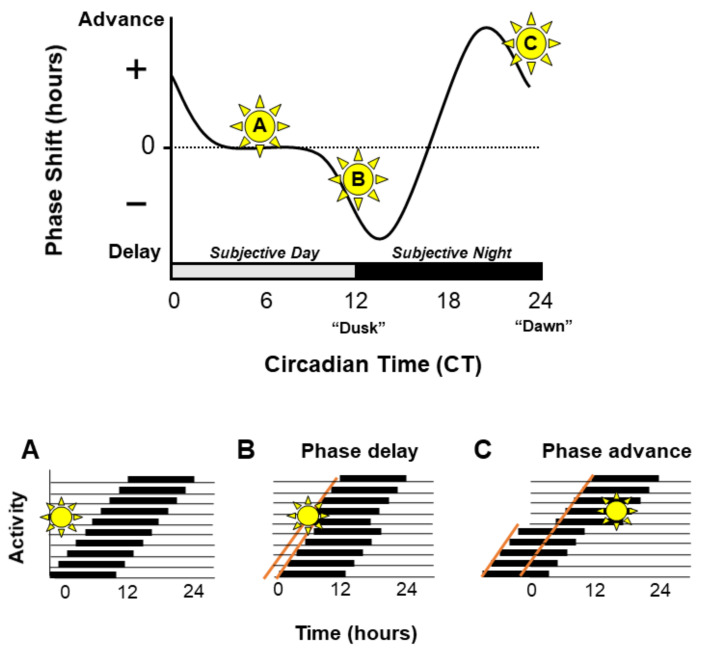
The phase response curve. The phase response curve demonstrates the effect of light exposure at different times of the circadian cycle on the phase of the circadian clock. Light delivered during the subjective day, the ‘dead-zone’ will have no effect on the phase of the clock (**A**). Light exposure during the early subjective night will lead to delays in the phase of the clock (**B**). Whereas light exposure at the end of the subjective night will lead to phase advances (**C**). This is demonstrated by representative actograms showing free running rest/activity rhythms (panels **A**–**C**); black bars represent periods of activity and black lines indicate rest.

Light is a powerful zeitgeber which can shift the phase of the circadian clock. However, the effects of light vary depending on the time of light exposure. Light detected during the twilight hours of dawn and dusk has the greatest impact, whilst light delivered during daytime has very little effect [6]. A key point is that the effects of light at dawn vs. dusk have opposite effects. Light exposure during the late night (dawn) will result in a phase advance of activity onset, therefore the animal will start its activity earlier the following day. Whereas light administered during the early night (dusk) will cause a phase delay in activity [7,8]. Such differential effects of light are represented as the “phase response curve” (PRC), an example of which is illustrated in Figure 2. Similar differential responses (PRCs) are seen across all organisms, including in both nocturnal and diurnal mammals. Although the size of the delays and advances vary between species, in all PRCs delays result from dusk light exposure, whilst advances occur after dawn light exposure. Overall entrainment is achieved by the summation of phase delays and phase advances.

## 3. Photoreception for Entrainment

In mammals, light input to the SCN is provided exclusively by the retina via the retinohypothalamic tract (RHT). Photoentrainment is abolished in blinded (enucleated) mammals [9,10,11], and in contrast to other vertebrates, mammals appear to lack extra-retinal photoreceptors [12]. However, the primary photoreceptors responsible for photoentrainment are not the rods and cones but a ‘third’ class of photoreceptor based upon a small number of photosensitive retinal ganglion cells (pRGCs) that utilise the photopigment melanopsin (OPN4) [13]. This was demonstrated by studies showing that circadian responses to light are intact in mice lacking rod and cone photoreceptors [14,15], but are impaired following ablation of the melanopsin gene, *Opn4* [16,17]. Nonetheless, it is only in the absence of all three photopigments (rod, cone and melanopsin) that circadian entrainment is completely abolished [18,19], demonstrating that rods and cones can contribute to photoentrainment in the absence of melanopsin.

For example, transgenic mouse studies have demonstrated that rod photoreceptors can partially mediate entrainment under scotopic (very dim) light levels [20,21]. This finding is consistent with the observation that rods drive electrical responses in light-sensitive SCN neurons under low light conditions [22]. In addition, studies have also shown that UV light detected by UV-sensitive cone photoreceptors (S-cones) will induce electrical responses in the SCN, along with phase-shifts in circadian activity rhythms [23,24]. Significantly, this input is sufficient for entrainment in the absence of melanopsin and rod photoreceptor signalling [25]. Finally, photoentrainment will occur following stimulation of the green-light sensitive M-cones. However, patterns of entrainment are not identical following OPN4, S-cone and M-cone stimulation, suggesting that different photoreceptor classes are “tuned” to the different features of the twilight transition. Indeed, recent evidence suggests that input from cones signals information on the spectral composition (colour) of light to the SCN, through activation of colour-sensitive neurons which can drive circadian phase and modulate entrainment [26,27]. This is thought to contribute to the detection of twilight transitions, during which there is a shift in the spectral environment to shorter wavelengths [28,29], which may act to support circadian entrainment under unreliable light intensity conditions. It is worth stressing that the melanopsin-expressing pRGCs are required to relay the rod and cone input to the SCN. If the pRGCs are specifically ablated in mice with intact rod and cone photoreceptors, photoentrainment is lost [30,31]. As a result, under normal circumstances, photoentrainment is achieved as a result of an integrated light input from all three photoreceptor classes, each encoding different aspects of the light environment at twilight.

## 4. Photosensitive Retinal Ganglion Cells (pRGCs)

The pRGCs represent a diverse population of cells consisting of five known subtypes (M1-M5) and a recently discovered sixth (M6), classified based on their anatomical and morphological differences [32,33]. They also exhibit distinct electrophysiological properties and mediate different light responses in addition to photoentrainment, including pupil constriction, sleep induction, masking, alertness and mood [12]. This appears to be achieved through diverse axonal projections to the SCN and multiple other brain targets. However, currently, the functional roles of each subtype remain poorly defined. A further complexity within these subpopulations has been demonstrated recently by the identification of distinct gene expression profiles by single cell transcriptomics [34]. Despite this diversity, retrograde tracing has identified the pRGC subtypes that mediate entrainment, with the majority of projections to the SCN attributed to M1 cells and a small proportion to M2 cells [35]. These subtypes vary in their expression of the two isoforms of melanopsin; M1 cells express both the short (OPN4S) and long (OPN4L) isoform, whereas M2 cells express only OPN4L [36,37]. In line with this, both isoforms have been shown to be required for photoentrainment [38]. Notably, it is a specific subset of just 200 M1 pRGCs that innervate the SCN, which are molecularly distinct to those projecting to other brain regions as they lack *Brn3b* expression [39]. Furthermore, gene expression patterns suggest that there are molecularly diverse subpopulations within this subset of M1 cells [34]. Further studies are required to determine whether these “molecular subpopulations” are sufficiently stable to constitute robust subdivisions of M1 pRGCs.

## 5. Molecular Photoentrainment of the Suprachiasmatic Nuclei (SCN)

**Figure 3:**
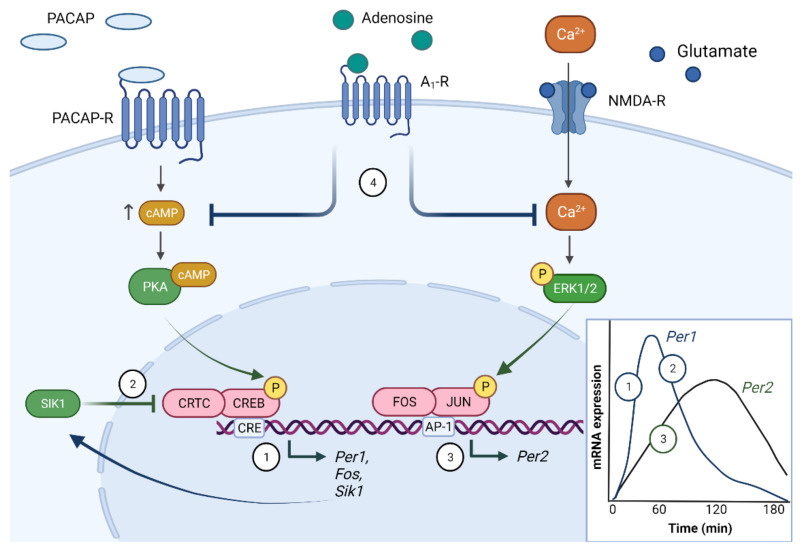
Molecular photoentrainment of the SCN. The light-induced release of glutamate and pituitary adenylate cyclase-activating polypeptide (PACAP) from the RHT nerve terminals leads to a rise in intracellular Ca^2+^ and cAMP levels in the SCN. These trigger a cascade of events including activation of protein kinase A (PKA), which activates the transcription factor cAMP response element-binding protein (CREB), together with co-activators such as CREB-regulated transcription coactivator 1 (CRTC1). This leads to the upregulation of CRE-driven genes, including the core clock component, *Per1* (1). In addition, *Sik1* is upregulated, which feedbacks on the CREB pathway by phosphorylation of CRTC1. This deactivates CRTC1 leading to a decline in CREB-induced gene transcription and therefore a decline in *Per1* expression (2). In parallel, the activation of ERK1/2 by Ca^2+^ influx leads to the upregulation of the immediate-early transcription factors JUN and FOS. These heterodimerise to form AP-1, which drives *Per2* transcription leading to an increase in *Per2* expression (3). Adenosine, which accumulates in the extracellular space during wakefulness, modulates these light-activated signalling pathways. Adenosine predominantly signals through the G_i_ (inhibitory) coupled A_1_ receptor in the SCN, which results in a decrease in intracellular cAMP and Ca^2+^ levels, and therefore a downregulation of the subsequent signalling events (4). Figure created with BioRender.com.

Retinal innervation of the SCN from the pRGCs is via the monosynaptic pathway of the RHT, the primary neurotransmitters of which are glutamate and pituitary adenylate cyclase-activating polypeptide (PACAP) [40]. Light-induced glutamate and PACAP release results in a rise in Ca^2+^ and cAMP levels, which in turn initiate a suite of kinase-based signalling cascades involving protein kinase A (PKA), protein kinase C (PKC), protein kinase G (PKG), mitogen-activated protein kinase (MAPK) and Ca^2+^/calmodulin-dependent protein kinase II (CaMKII) [41]. The primary mechanism for entrainment is considered to be the activation of the transcription factor cAMP response element-binding protein (CREB), through phosphorylation at Ser133 and Ser142 [42,43], which then modulates the transcription of the clock genes *Per1*/*Per2* (Figure 3). The light-induced upregulation of *Per1*/*2* adjusts the TTFL which shifts the phase of the clock into alignment with the external light/dark cycle. The phase at which light-induced *Per1*/*2* upregulation occurs will determine the direction of the phase shift. As outlined above, dawn light exposure will lead to a phase advance in the clock, whereas dusk light exposure will lead to a phase delay [7,44]. Whilst the molecular pathways underlying both delays and advances are broadly similar and rely on cAMP-CREB transcription, there are key differences. *Per1*/*Per2* expression within the SCN is rhythmic, overall both genes are elevated during the day and low at night. Light at dawn acutely increases *Per1* transcription thus advancing the onset of the rhythm in *Per1*, whilst light at dusk increases *Per2* stability, thus delaying the offset of the *Per2* rhythm [45]. Furthermore, Ca^2+^ release by ryanodine receptors only correlates with phase delays [46], whilst the increase in cyclin GMP (cGMP) and activation of cGMP-dependent kinase (PKG) characterises phase advances [47,48]. Accordingly, sildenafil, which inhibits cGMP-specific phosphodiesterase 5, enhances photic phase advances in hamsters [49].

The light-regulated transcriptome comprises hundreds of genes, including immediate early genes (IEGs) and CREB target genes [50,51]. Undoubtedly, the most important of these are *Per1* and *Per2*, but other elements of this transcriptome also play an important role. IEGs are part of the transcriptional network controlling *Per1*/*2* transcription. *Per1* is rapidly upregulated within 30 min of light exposure, whereas *Per2* induction is slower with a timeframe of several hours [44]. Recent evidence suggests this is due to the induction of *Per1* by CREB, and the induction of *Per2* by a parallel pathway involving the activation of ERK1/2 by Ca^2+^ influx, leading to upregulation of the immediate-early transcription factors JUN and FOS [52]. These then heterodimerise to form AP-1, which drives *Per2* transcription and other genes containing AP-1 response elements (Figure 3). In addition, other light-regulated IEGs have also been shown to regulate photic input; *Npas4* is upregulated by light within the SCN and regulates the expression of multiple genes including *Per1*. *Npas4*^−/−^ animals show unstable photic entrainment and reduced phase-shifting in response to light [53]. In addition, the light-regulated transcriptome also includes multiple kinases and phosphatases, such as *Sik1* and *Dusp4* [54], which are part of the cascade regulating light responses within the SCN, and thus photic entrainment. It is clear that whilst the role of a notable fraction of the light-regulated transcriptome can be linked to photoentrainment, the functional role of much of this light-regulated transcriptome remains unknown.

## 6. Gating the Light Sensitivity of the Clock

The molecular light responses of the SCN are regulated throughout the day/night cycle such that the SCN responds to resetting signals in a time-dependent manner. Central to this is the gating of the light-induced phosphorylation of CREB, which is limited to nocturnal hours and, therefore, light exposure during the subjective day has little or no effect upon *Per1*/*2* expression [42,55]. The signalling pathways mediating this are currently poorly understood, however vasoactive intestinal peptide (VIP) is thought to be necessary for photic gating [56], and recent evidence has demonstrated that it acts via DUSP4, a negative regulator of ERK1/2 [54]. VIP-expressing neurons receive direct innervation from the retina, and VIP signalling is necessary for maintaining the synchronisation of circadian oscillations across the SCN network [57,58]. Interestingly, another negative regulator of ERK1/2, the Ras-like G-protein DEXRAS1, has also been shown to be necessary for photic gating. Transgenic mouse studies found that loss of DEXRAS1 altered the phase-dependent light sensitivity of the clock, with daytime light pulses inducing phase shifts in activity [59,60]. Therefore, inhibitors of ERK1/2 appear to be important for blocking light-induced responses of the SCN during the day. Overall, this gating of the SCN response to light acts to limit the impact of transient fluctuations in the light environment on the clock, which may otherwise result in desynchronization with the light/dark cycle.

The effect of light on the circadian system is not equal at all times, it varies with both time of day (see discussion on the PRC above) and physiological state. The shape of the PRC varies with the temporal niche occupied by the organism, nocturnal animals typically display large delays at dusk, but smaller advances at dawn, whereas the reverse is true for diurnal animals [6]. Furthermore, sleep history impacts photic responses; sleep deprivation reduces both light-induced electrical activity within the SCN [61] and the size of resulting phase shifts in mice [62,63] and humans [64], although exceptions exist; sleep deprivation potentiates photic phase shifts in the diurnal rodent, *Arvicanthus ansorgie* [65]. The molecular mechanisms underlying these observations can in part be attributed to adenosine signalling. Adenosine, as a by-product of ATP metabolism, accumulates in the extracellular space in a manner correlating with wake time [66,67]. It then activates signalling from adenosine receptors, which are predominantly the A_1_ (G_i_-coupled) and A_2A_ (G_s_-coupled) receptors in the SCN. Such G protein-coupled receptor (GPCR) signalling converges on the same pathways that are activated by light, specifically cAMP-CREB and Ca^2+^-ERK1/2-AP-1, thus resulting in *Per1/2* upregulation. As the A_1_ receptor is the dominant form within the SCN, elevated adenosine, as occurs following sleep deprivation, inhibits the effect of light on the circadian system (Figure 3), whilst adenosine A_1_/A_2A_ antagonists enhance photic responses [52]. Caffeine, which has A_1_/A_2A_ antagonism properties, both enhances photic shifts in humans and counters the effects of sleep deprivation on the clock in mice [52,63,68,69]. This pathway provides a molecular framework by which the major drives that control sleep/wake transitions interact, namely the circadian (Process C) and homeostatic (Process S) drives [70,71], thus allowing sleep history to shape photic entrainment processes [52].

## 7. Buffering Photoentrainment

Another key feature of photoentrainment is that re-entrainment to a shifted light/dark cycle is slow, taking several days. It is typically limited to one hour per day in most mammals [72]. This mechanism acts to limit the size of phase shifts to “buffer” the circadian system from extreme shifts which may result in internal desynchronization. This buffering is thought to be due to the constrained induction of *Per1* by light. After *Per1* mRNA is induced by light it peaks at 1 h before returning to baseline after 3 h [73]. This suggests that CREB-mediated transcription is curtailed shortly after its induction by light. This inhibition has been shown to be driven by the CRTC1-SIK1 pathway, whereby light induces transcription of CREB-regulated transcription coactivator 1 (CRTC1), which then co-activates CREB inducing expression of *Per1* and *Sik1* [50]. SIK1 then phosphorylates CRTC1 leading to its deactivation and, therefore, the decline in *Per1* transcription (Figure 3). The net result is that SIK1 acts as a “brake” on the induction of *Per1*. This has been demonstrated at a behavioural level through RNAi knockdown of *Sik1* in the SCN of mice. Following a 6 h advance of the light/dark cycle there was a rapid re-entrainment in *Sik1* knockdown mice compared to controls [50]. This rapid re-entrainment is also observed in transgenic mice expressing a catalytically inactive version of SIK1 [74].

In addition, the activation of *Per1* expression by CLOCK:BMAL1 binding at the E-box response element is also subject to modulation to limit light-induced *Per1* expression. Transgenic mouse studies suggest that the transcriptional repressor, ID2, interacts with CLOCK:BMAL1 to limit *Per1* induction and circadian responses to light; in the absence of *Id2*, mice show rapid re-entrainment to phase delays which is accompanied by elevated light-induced *Per1* expression [75,76]. ID2 is thought to act by sequestering CLOCK and BMAL1 to the cytoplasm [77]. Interestingly, recent evidence suggests that another member of the same family, ID4, is also involved in photic entrainment but with opposing effects to ID2 [78].

The regulation of PER stability is another target for buffering the effects of light on the clock. For example, there is evidence that another member of the SIK family, SIK3, may participate in this process. In vitro experiments have shown that phosphorylation of PER2 by SIK3 regulates the abundance of PER2 by promoting its degradation [79]. Transgenic mouse studies suggest that this is important at the behavioural level, with *Sik3*^−/−^ mice exhibiting significant phase delays and lengthened circadian periods. This suggests that the two SIK isoforms regulate circadian entrainment via distinct substrates and signalling pathways. However, different SIK mouse models yield conflicting results; gain of function mutants of *Sik1* [80] or *Sik3* [81] do not show deficits in circadian behaviour, however gain of function kinase models cannot fully capture a kinase’s function. Such models cannot describe how each kinase is endogenously regulated, and the context in which they are activated, as they are “always on”.

Moreover, the SIK family of kinases is also now known to regulate sleep through a pathway involving phosphorylation of synaptic proteins [82]. Mice with a gain of function mutation in *Sik3* have a constitutively elevated sleep need, associated with hyperphosphorylation of proteins at the synapse [81,82]. Such protein phosphorylation is also seen following sleep deprivation [83], suggesting that the action of SIK on synaptic proteins provides a molecular substrate for sleep. The regulation of SIK1 by light appears also to regulate a similar set of substrates, thus leading to the induction sleep in mice [74]. Thus, it appears that the SIK family of kinases play an important role in transducing both environmental and physiological signals to both the sleep and circadian systems in parallel in order to adjust sleep/wake timing.

PER stability is also modulated by the key clock component casein kinase 1 (CK1), which phosphorylates PER1, PER2 and PER3 to target these proteins for degradation [84,85]. This action is thought to underpin the role of CK1 as a regulator of the speed of the TTFL [86,87]. Furthermore, lack of CK1ε leads to faster re-entrainment following both phase advances and delays of the light/dark cycle [88]. This suggests that CK1ε limits the light-induced accumulation of PER in the SCN, which in turn limits the size of behavioural phase shifts.

In addition to the cell autonomous molecular clockwork, light can directly affect circuit level properties of the SCN. Much of the robustness of SCN rhythms is attributed to the tight coupling between the cells of the SCN through the neurotransmitters arginine vasopressin (AVP), VIP and GABA. These are expressed by discrete cell types within the SCN, with AVP restricted largely to the shell region and VIP to the core, where input from the RHT is localised. Light input activates VIP neurons, which in turn activate other regions including the AVP neurons and dorsomedial hypothalamus, thus regulating circadian rhythms of rest and activity [54,89]. The ablation of vasopressin V1a and V1b receptors or their blockade resulted in reduced synchrony amongst the SCN cells and more rapid entrainment to a shifted light/dark cycle [90]. Whilst AVP/VIP-mediated coupling confers great stability on SCN rhythms, it is also believed to confer rigidity, or inertia to resetting. The temporary loss of this coupling would allow the individual neurons to unlock their phase coupling and, therefore, achieve greater shifts, as illustrated by the studies above.

## 8. Concluding Remarks

It is clear that the molecular basis of photic entrainment in the SCN is complex. Whilst the classical cAMP-CREB-PER pathway plays a central role, recent advances have identified additional key signalling pathways and regulators that act in parallel. Collectively, these pathways regulate the phase-shifting effects of light on the clock, thus making circadian entrainment a gradual and carefully controlled process. Significantly, major progress has been made in our understanding of the interaction between sleep and circadian entrainment, which has revealed molecular cross-talk between these two processes and further strengthened their close bidirectional relationship.

## Data Availability

Not applicable.
